# Comparative Y-chromosome analysis among Cypriots in the context of historical events and migrations

**DOI:** 10.1371/journal.pone.0255140

**Published:** 2021-08-23

**Authors:** Irene Moutsouri, Anna Keravnou, Panayiotis Manoli, Stefania Bertoncini, Kyriaki Michailidou, Vasilis Christofi, Stavroulla Xenophontos, Marios A. Cariolou, Evy Bashiardes

**Affiliations:** 1 Cyprus School of Molecular Medicine, The Cyprus Institute of Neurology and Genetics, Nicosia, Cyprus; 2 Department of Cardiovascular Genetics and The Laboratory of Forensic Genetics, The Cyprus Institute of Neurology and Genetics, Nicosia, Cyprus; 3 Department of Biology, University of Pisa, Pisa, Italy; 4 Biostatistics Unit, The Cyprus Institute of Neurology and Genetics, Nicosia, Cyprus; Universitat Pompeu Fabra, SPAIN

## Abstract

Y-chromosome analysis provides valuable information regarding the migration patterns of male ancestors, ranging from the Paleolithic age to the modern humans. STR and SNP genotyping analysis provides data regarding the genetic and geographical ancestry of the populations studied. This study focused on the analysis of the Y-chromosome in Maronite Cypriots and Armenian Cypriots, who came to the island as a result of different historical events. The aim was to provide information on the paternal genetic ancestry of Maronites and Armenians of Cyprus and investigate any affinity with the Greek Cypriots and Turkish Cypriots of the island. Since there is limited information in the current literature, we proceeded and used 23 Y-chromosome STRs and 28 Y-chromosome SNPs to genotype 57 Maronite Cypriots and 56 Armenian Cypriots, which were then compared to data from 344 Greek Cypriots and 380 Turkish Cypriots. All samples were assigned to eight major Y-haplogroups but the most frequent haplogroup among all Cypriots is haplogroup J in the major subclade J2a-L559. The calculated pairwise genetic distances between the populations show that Armenian Cypriots are genetically closer to Greek and Turkish Cypriots compared to Maronite Cypriots. Median Joining Network analysis in 17 Y-STR haplotypes of all Cypriots assigned to J2a-L559, revealed that Cypriots share a common paternal ancestor, prior to the migration of the Armenians and Maronites to Cyprus, estimated in the Late Bronze Age and Early Iron Age.

## Introduction

Cyprus is the third largest island in the Mediterranean Sea neighboring with Lebanon, Turkey, Syria, Israel, Egypt and Greece. Because of its geographical location, the country has a long history of governance and several cultures which have influenced, not only the ethnic composition of its population but also its culture and its lifestyle [1]. This indicates that the genetic ancestry of Cypriots will vary, since many individuals from various populations resided in Cyprus. Two major population groups comprise the community of Cyprus, the Greek Cypriots (GCys) (77%) and the Turkish Cypriots (TCys) (18%). The remaining proportion of the population belongs to a number of individuals who settled in Cyprus and in 1960 were recognized as separate religious groups. These include the Maronite Catholics, Armenian Orthodox and Latin Roman Catholics with individuals approximating 7000, 3500 and 900, respectively [2], from a total population of 840,407 (population census 2011, statistical services, Republic of Cyprus).

Maronites are the largest of the three religious groups. The first Maronites and Armenians came to the island during the 7^th^ and 6^th^ century, respectively [3]. Maronites settled in Cyprus in three different waves. The first appearance of Maronites in Cyprus is documented to follow religious clashes in Syria and Lebanon in 686 AD with the island being in close proximity, it was considered a safe place to settle. The second wave occurred in 938 AD after the destruction of Saint Maron’s Monastery in Apamea, Syria. The last big wave of Maronites settling in Cyprus was recorded in the late 12^th^ century, when the King of Jerusalem, Guy de Lusignan, acquired Cyprus, during the Frankish Era in 1192 [4].

The first arrival of Armenians in Cyprus is estimated to have occurred during the Byzantine Era, but there was a continuous influx to the island since 578 AD [5]. The Byzantine General, Maurice the Cappadocian, captured approximately 10000 Armenians in Arzanene Armenia, with more than 30% fleeing to Cyprus and subsequently establishing communities [5]. During the reign in Cyprus of Armenian descended Emperor Heraclius (the period 610–641 AD) and during the pontificate of Catholicos Hovhannes Odzents (the period 717–728 AD), a number of Armenians arrived on the island for political and commercial reasons [5]. Cyprus survived the Arab raids in 965 AD, so more Armenians considered the island a safe place to settle. Furthermore, a big wave came from Western Europe, Cilicia and the Levant during the Latin era in 1192 AD [5]. A series of events resulted in the arrival of Armenians in Cyprus; in 1267 AD after the fall of Jerusalem, in 1291 AD after the fall of Acre and in 1335 AD after the Saracen attacks on Cilicia. With the passage of time, more refugees arrived on the island amounting to around 10000 from Cilicia, Smyrna and Constantinople as a result of massive deportations and the Armenian genocide by the Ottomans in 1915 [5].

The human Y-chromosome is a great tool for the analysis of the migration pattern of paternal lineages through the years. Previous studies have been undertaken to determine the haplogroups of the Maronites (including Maronite Cypriots) and Armenians but not of Armenian Cypriots [6–8]. So far, no studies have compared the ancestry of Armenian Cypriots and Maronite Cypriots with the Greek Cypriots and Turkish Cypriots.

Maronite Cypriots originated mainly from Lebanon [4]. A study regarding the Lebanese population, identified that haplogroups J-M267, J-M172, and E-M35 were observed at a high frequency but others such as G, I, K2, L and R1b were observed at a lower frequency [6]. Maronites in Lebanon are Christians and it is believed that with the migration and isolation within Lebanon, they acquired affinity from Byzantine and Muslim cultures [6]. Haber *et al* (2010) analyzed the ancestry of 42 Maronite Cypriots, which were included in the Lebanese Maronite population. Between all populations, haplogroups J-M267, J-M172, and E-M35 were the most frequent, with the most common haplogroup in Maronite Cypriots being E-M35 [6].

Previous studies have been performed for the determination of the origin of Armenians [7, 8]. A study in four distinct Armenian populations was performed to search first for potential differences in the Neolithic and Indo-European populations and then, the consequences of Armenian history on patrilineal genetic structure [8]. Several haplogroups were identified within the four populations, which were analyzed, but the R-M207 and J-M304 haplogroups seem to be the most dominant; within the R and J Υ-haplogroups. The largely Near Eastern lineage R1b1b1*L23 and within the J-M304, the lineage J2a*-M410 are the most dominant [8]. Another study found that Armenians, Jews, Druze and Lebanese Christians have a similar genetic background and show genetic continuity with the Caucasus [7]. With respect to the ancestry of Armenian Cypriots, so far, no study has analyzed their phylogenetic background.

The present study aimed to determine whether paternal lineages of Maronite Cypriots and Armenian Cypriots had affinity with the Greek Cypriots and Turkish Cypriots. A comparative analysis with other available population data also investigated the level of admixture and influence from other neighboring as well as more distant populations in the context of historical events and concomitant population migrations. The genetic data which were used from other studies for the genetic calculations were Y-Short Tandem Repeats (Y-STRs) and Y-Single Nucleotide Polymorphisms (Y-SNPs) [8–12].

## Materials and methods

### Sample collection and DNA extraction

The design and execution of this project fulfilled all the personal data protection requirements stipulated by the General Data Protection Regulation (GDPR 2016/679). The study was approved by the Cyprus National Bioethics Committee (CNBC). Informed consent was given by all interested participants. Inclusion criteria were as follows: unrelated males, who were 18 years old or above, born in Cyprus, with a Maronite or Armenian paternal ancestry.

A total of 57 and 56 unrelated Maronite Cypriots and Armenian Cypriots, respectively, participated in the study. Unfortunately, due to the SARS-CoV-2 pandemic, a decision was taken to stop further recruitment of individuals.

DNA extraction was carried out using a QIAGEN BioRobot Universal (BRU) with QIAamp 96 DNA Swab BioRobot Kit or by using QIAamp DNA investigator kits from QIAGEN [13]. A reagent blank was used in all DNA extractions for quality control.

### Y-chromosome genotyping and haplogroup prediction

The extracted total nuclear DNA was quantified using an internally validated published method running on the AB-7500 RT PCR System and set up using the BRU. Automated Y-STR PCR set-up followed using 500pg DNA template. The Y-STR amplification kit used was PowerPlex Y23 (Promega). All relevant controls were included in the STR PCR set-up to verify the validity of the results generated. Following PCR, aliquots of amplified products were run on the AB-3130xl capillary electrophoresis genetic analyzer (Applied Biosystems). Once the run was completed, the data were analyzed via GeneMapper ID V3.2 using the analysis parameters previously imported for this Y-STR system. Data have been submitted to the online Y-STR Haplotype Reference Database (YHRD) (https://yhrd.org/) with the accession numbers: YA004717, YA004718. Y-23-STR haplotypes of all individuals were imported individually, in the NEVGEN predictor tool (https://www.nevgen.org/) to predict their respective haplogroups [14]. After sorting, a total of nine major haplogroups (E, F, G, I, J, L, Q, R, T) and their respective subclades for both Maronite and Armenian male Cypriots (n = 113) were predicted.

### Y-haplogroup subclade determination

Based on the aforementioned, the 28 Y-SNP reference IDs, for each haplogroup subclade and their position on the Y-chromosome, were identified using the Single Nucleotide Polymorphism Database of the National Center of Biotechnology Information (NCBI) (https://www.ncbi.nlm.nih.gov/) and the International Society of Genetic Genealogy (ISoGG) (https://isogg.org/) (Table 1). Two methods (MassArray assay and Sanger sequencing) were used to assign the haplogroup subclade for each sample in the current dataset. In Table 1, the 18 highlighted cells represent the Y-SNPs, which were used in the iPLEX Genotyping MassArray assay. The PCR and iPLEX Genotyping MassArray extension primers for each of the 18 SNPs were designed using the Assay Design Suite software by Agena Bioscience. Sequence information from the other 10 SNPs, (Table 1; non-highlighted cells), was used to define the haplogroup subclade following, targeted, Sanger sequencing of the Y-chromosome. The primers and sequences of all 28 Y-SNPs used are available in S1 Table.

**Table 1:** Y SNPs used for Y-haplogroup subclade assignment.

Y-haplogroup	rs number	Marker	ISOGG
E1a	rs2032617	M132	G>T
E1b1b1	rs375228668	M35.1	G>C
E1b1b1a1	rs368977028	M78	C>T
E1b1b1a1b2	rs368511174	V22	T>C
E1b1b1b2a1	rs371143248	M123	C>T
E1b1b1b2a1a~	rs373666971	M34	G>T
G2	rs4116820	P287	G>T
G2a1	rs750938499	Z6552	G>A
G2a2	rs561766566	M3308	C>G
I	rs2032597	M170	A>C
I1	rs17316597	M450	G>A
I2	rs35547782	L68	C>T
J	rs17315835	P209	T>C
J1	rs9341313	M267	T>G
J1a	rs375539978	Z2392	T>A
J2	rs2032604	M172	T>G
J2a	rs373707621	L559	A>G
J2b	rs3903	M12	G>T
L1b	rs376844020	L655	C>T
Q2	rs780551375	F1280	C>A
R	rs2032658	M207	A>G
R1a	rs17250535	M420	T>A
R1b	rs9786184	M343	C>A
R1b1a	rs1358368	L389	C>G
R2	rs372157627	M479	C>T
T	rs20320	M184	G>A
T1	rs756850544	PF5641	G>A
T2	rs780245732	PH110	G>T

### Haplotype (Υ-STR) based analysis

Genetic data (Y-STRs) from Greek Cypriots and Turkish Cypriots obtained from previous studies [15, 16] were analysed herein. Genetic diversity (He) for Greek Cypriots, Turkish Cypriots, Maronite Cypriots and Armenian Cypriots was calculated using Haplotype analysis Microsoft Office Excel Add-in [17]. Calculations were performed using 22 Y-STR loci, excluding the DYS458 locus due to the microvariants observed. The range of ‘He’ is from 0 to 1, with 0 indicating no diversity within the population.

The Y-STR Haplotype Reference Database (YHRD) is a searchable worldwide database of Y-STR Haplotypes (https://yhrd.org/). YHRD performs an assessment of male population stratification among worldwide populations using Y-STR and Y-SNP frequency distribution [18]. Pairwise genetic distances were calculated between Greek Cypriots, Turkish Cypriots, Maronite Cypriots and Armenian Cypriots, using Spatial Pattern Analysis of Genetic Diversity (SPAGeDi) software and YHRD online tool (https://yhrd.org/) [19, 20]. Rst values range between the values 0 and 1, where, 0 indicates no differentiation and 1 indicates complete differentiation. The two software were used in order to confirm the genetic correlation and statistical significance between populations. One important step before the calculation of the statistics using SPAGeDi, was the creation of the appropriate input data file with the population data in order to facilitate the analysis and identify statistically significant results. Calculations in SPAGeDi software were performed under population level, according to the global R statistics and pairwise Rst (F statistics analogue based on allele size). There are three levels of differentiation when considering genetic distances. Values between 0–0.05 indicate low differentiation, the range between 0.05–0.15 indicates moderate differentiation and values greater than 0.15 indicate high levels of differentiation [21]. The statistical significance level of p-values was defined using 10,000 permutation tests in both software. P-values were calculated to assess the differentiation among populations of interest. More precisely, p-values ≥ 0.05 show no significant differentiation, in contrast with values <0.05 which show significant differentiation. Permutation tests in combination with the genetic distances provide more accurate results regarding genetic differentiation [22]. Pairwise comparisons can describe relatedness or differentiation between the populations. Permutation tests under population level are performed using microsatellite allele sizes to test if, the mutation rate is sufficient to affect the genetic structure [19]. Multidimensional scaling plot (MDS) was created using the YHRD Online tool, which represents the genetic correlation between the populations of interest, based on the calculated genetic distances. The MDS plot is based on Kruskal’s non-metric multidimensional scaling [20].

A dataset for the Y-STR analysis was created, using the data of all Cypriots and the available population data from countries where Maronites and Armenians may have originated before coming to Cyprus. For the Turkish Cypriot population, only 17 Y-STR loci were available from a previous study [16], therefore, haplotypes of the other populations (Greek Cypriots, Maronite Cypriots and Armenian Cypriots, Armenians, Egyptians, Syrians, Lebanese, Turks) were adjusted to the 17 loci. The 17 loci used were the following: DYS389 I, DYS448, DYS389 II, DYS19, DYS391, DYS438, DYS437, DYS635, DYS390, DYS439, DYS392, DYS393, DYS458, DYS385a, DYS385b, DYS456 and YGATAH4. It was observed that locus DYS458 contained many duplicated and triplicated intermediate values (i.e alleles), thus, the values for the particular locus were excluded from the genetic distance calculations. Using the R programming and R studio, contour maps were created according to the pairwise genetic distances among populations.

Median Joining Network (MJN) diagrams were constructed using the software Network 4.6.1.6 Fluxus Engineering [23–25], representing the phylogenetic relationships of the common loci of individuals from all populations. The phylogenetic network can visualize evolutionary similarities. The aim of the software is to reconstruct the shortest possible and least complex phylogenetic trees from a given dataset [24]. There is a star contraction option, which is used to simplify a network or to identify population expansion events. The star contraction algorithm can identify clusters among the haplotypes and create a contraction which looks like a star [26]. Using the constructed star, time to the most recent common ancestor (TMRCA) can be estimated. The MJN was created based on the 17 Y-STR loci of all Cypriot Y-haplotypes, which were assigned to haplogroup J2a that was represented in all the Cypriot samples of this study, with percentages above 20. For the time estimation, the following were used: Y-haplotypes were entered into the Network software to calculate the default time in years for 1 mutation. This was calculated to 1 mutation every 20180 years. When this was applied to the Network software, age in years was calculated to be 168166 for the first expansion event and 178836 for the second expansion event. The ancestral node age, 3070 years ago, was found from the existing literature [27]. The time in years for 1 mutation for each expansion event was calculated using the formula:ancestral node age (i.e. 3070) / age in years (i.e. 168166 or 178836) x default time in years for 1 mutation (i.e. 20180) [23, 24, 28]. An additional method was used for the expansion time calculation for the two expansion events. More specifically, using the formula: mutation rate (i.e. 0.0025) x locus (i.e. 17) x generations (i.e. 25) [15, 29, 30], a value of 0.0017 was obtained. Then 1/0.0017 = 588 provided the time in years for 1 mutation for both expansion events. Subsequently, all the four calculated values for the time in years for 1 mutation, were applied in the Network software to provide an estimate for the expansion time for each of the two events. Confidence Intervals of the TMRCA were calculated using the Rho statistic method of the Network software (Age in years +/- Standard Deviation in years [24, 28, 31].

### Haplogroup based analysis

Each sample in each population was assigned to a Y-haplogroup. Y-haplogroup frequencies were calculated for each population and then combined analysis was followed for the comparison between the haplogroups in all populations. Mappies were created using Υ-haplogroup frequencies and R programming, which represent the proportion of each haplogroup in a pie chart, located to the centroid of each country [32]. Y-paths of each haplogroup were created, using the literature, in combination with the previous publications with relevant historical events. The Y-paths represent the distribution and expansion of each haplogroup in the different regions and countries. Some packages used in R studio were: doBy, ape, ggmap, rworldmap and ggplot2 [32, 33]. Based on the Y-paths and the countries which are included in each path, additional pairwise genetic distances were calculated among those countries and all Cypriot haplotypes, using YHRD online tool.

## Results

### Intra-population analysis

The aim of the study was to find the genetic differentiation between all Cypriots, Greek, Turkish Cypriots, Armenian Cypriots and Maronite Cypriots. In the current study, 57 and 56 Y-STR profiles from Maronite and Armenian Cypriots (S2 Table), respectively, were compared initially with Greek Cypriots and Turkish Cypriots [15] and then with the Y-STRs of the countries, from which, Maronites and Armenians may have originated before coming to Cyprus [8–12]. Thirty eight out of the 57 Y-STR haplotypes were unique among the Maronite Cypriot samples and 47 out of the 56 Y-STRs were unique among the Armenian Cypriot samples (S3 Table). The values of genetic diversity in both populations (i.e. Maronite Cypriots and Armenian Cypriots) were close to 1 (S3 Table) indicating high diversity and genetic variability within each population. The “He” value of Maronite Cypriots was slightly lower compared with the “He” of Armenian Cypriots and this is explained by the number of unique haplotypes within the Maronite Cypriots, which was lower compared to the Armenian Cypriots. Consequently, there is an agreement between the low number of shared haplotypes and the high value of genetic diversity.

Both software analysis results were concordant with respect to the genetic correlation between Cypriots (Table 2). Maronite Cypriots show moderate genetic differentiation with Armenian Cypriots, Greek Cypriots and Turkish Cypriots. In contrast, Armenian Cypriots showed low genetic differentiation with Greek Cypriots and Turkish Cypriots. Maronite Cypriots and Armenian Cypriots showed moderate genetic differentiation between each other, but their correlation was greater than the correlation between Maronite Cypriots with Greek Cypriots and Turkish Cypriots. It is observed that the genetic differentiation value between Maronite Cypriots and Greek Cypriots and the value between Maronite Cypriots and Turkish Cypriots are very close (Table 2). The same correlation is shown also in the values between Armenian Cypriots with Greek Cypriots and Armenian Cypriots with Turkish Cypriots. This could be explained by the very low genetic differentiation between Greek Cypriots and Turkish Cypriots and that they share a common ancestor [15].

**Table 2:** Pairwise genetic distances (Rst values) between all Cypriots.

Population	Maronite Cypriots (n = 57)	Population	Armenian Cypriots (n = 56)
**SPAGeDi Analysis**		**SPAGeDi Analysis**	
Cyprus [Turkish Cypriots]	0.0521	Cyprus [Turkish Cypriots]	0.0226
Cyprus [Greek Cypriots]	0.0581	Cyprus [Greek Cypriots]	0.0255
Cyprus [Armenian Cypriots]	0.1069	Cyprus [Maronite Cypriots]	0.1069
**YHRD Analysis**		**YHRD Analysis**	
Cyprus [Greek Cypriots]	0.0756	Cyprus [Turkish Cypriots]	0.0396
Cyprus [Turkish Cypriots]	0.0853	Cyprus [Greek Cypriots]	0.0472
Cyprus [Armenian Cypriots]	0.1812	Cyprus [Maronite Cypriots]	0.1812

### Inter-population analysis

Pairwise genetic distances between Maronite Cypriots and Armenian Cypriots and populations from other countries were calculated, using two different software, SPAGeDi and YHRD. For the calculation, available datasets from Cyprus, Armenia, Egypt, Lebanon, Syria and Turkey were used [8–12, 15]. Rst values and MDS were calculated using the YHRD online tool [20]. According to the SPAGeDi results, Maronite Cypriots showed low genetic differentiation with the Lebanese population (Table 3). This was the only population with which Maronite Cypriots were genetically close. Moderate genetic differentiation was observed between Maronites and Egyptians, Israelis and Syrians with values between the ranges 0.05–0.15 (Table 3). The greatest genetic distance was demonstrated between Maronite Cypriots and Armenians. According to the two software used for the calculation of genetic distances, SPAGeDi showed a moderate genetic correlation between Maronites Cypriots and Syrians, while YHRD showed a great differentiation between them (Table 3). The different genetic data and the different algorithms [34, 35] used for the calculation of the genetic distances account for the disparity between the Rst values from the two software. The input data for the YHRD were available datasets from the YHRD database. The input data for the SPAGeDi were available datasets from previous publications. The algorithm used in YHRD was based on Excoffier et al. 1992 [34] compared with the algorithm used in SPAGeDi which was based on Nei’s standard genetic distance [35].

**Table 3:** Pairwise genetic distances (Rst values) between Maronite and Armenian Cypriots and countries from which they originated before coming to Cyprus.

SPAGeDi Software Analysis			
**57 Maronite Cypriots compared with**	Rst Values)	**56 Armenians Cypriots compared with**	Rst Values)
Lebanon [Lebanese]	0.0246	Syria [Syriacs]	0.0076
Cyprus [Turkish Cypriots]	0.0521	Turkey [Turkish]	0.0146
Cyprus [Greek Cypriots]	0.0581	Cyprus [Turkish Cypriots]	0.0226
Egypt [Egyptians]	0.1060	Cyprus [Greek Cypriots]	0.0255
Cyprus [Armenian Cypriots]	0.1069	Lebanon [Lebanese]	0.0308
Syria [Syriacs]	0.1405	Armenia [Armenians]	0.0703
Turkey [Turkish]	0.1634	Cyprus [Armenian Cypriots]	0.1069
Armenia [Armenians]	0.3887	Egypt [Egyptians]	0.1179
YHRD Software Analysis			
**57 Maronite Cypriots compared with**	Rst Values)	**56 Armenians Cypriots compared with**	Rst Values)
Egypt [Egyptians]	0.0515	Turkey [Turkish]	0.0100
Cyprus [Greek Cypriots]	0.0756	Armenia [Armenians]	0.0206
Lebanon [Lebanese]	0.0767	Syria [Syriacs]	0.0243
Northern Israel, Israel [Druze]	0.0798	Lebanon [Lebanese]	0.0298
Israel & Palestinian Authority Area [Arab]	0.0825	Northern Israel, Israel [Druze]	0.0393
Cyprus [Turkish Cypriots]	0.0853	Cyprus [Turkish Cypriots]	0.0396
Israel & Palestinian Authority Area [Samaritan]	0.1156	Cyprus [Greek Cypriots]	0.0472
Cyprus [Armenian Cypriots]	0.1812	Israel & Palestinian Authority Area [Arab]	0.1175
Turkey [Turkish]	0.1987	Egypt [Egyptians]	0.1369
Syria [Syriacs]	0.2623	Cyprus [Maronite Cypriots]	0.1812
Armenia [Armenians]	0.2638	Israel & Palestinian Authority Area [Samaritan]	0.2206

Armenian Cypriots were very close genetically with all Cypriots, based on the results of both software (Table 3). The lowest genetic distance observed was between Armenian Cypriots, Syrians, and Turks. The p-value of the genetic distance between Armenian Cypriots and Turks was the only value above the 0.05 limit. This confirms that there was no significant differentiation between the two populations. Low genetic differentiation was observed also between Armenian Cypriots and Armenian, Lebanese and Israeli populations. The genetic distances between Armenian Cypriots, Egyptians and Israelis and those under the Palestinian Authority (Arabs) show moderate differentiation between them (Table 3). The greater genetic distances were between Armenians, Samaritans and Maronite Cypriots with values above the 0.15 threshold.

**Fig 1:**
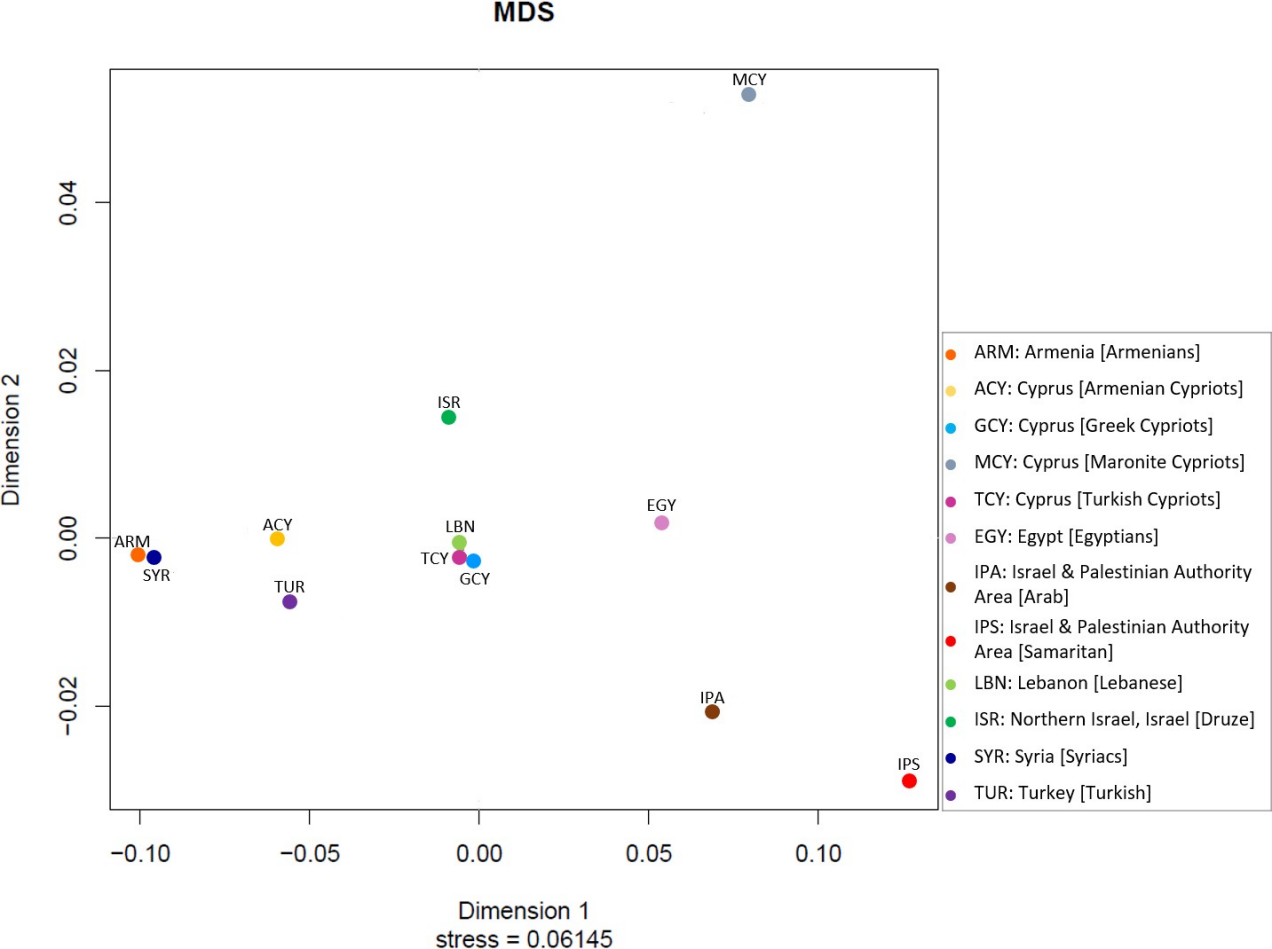
Multidimensional scaling plot between Cypriots and populations of interest. The measure of goodness of fit in MDS is called stress and stress values below 0.15 represent a good fit of the Rst values of the genetic distances in the plot. The value is 0.06145 confirming the good fit of the Rst values between the populations in the plot.

The genetic correlations between the populations are shown in the MDS plot in Fig 1. The difference between the genetic correlation of Maronite Cypriots and Armenian Cypriots with Greek Cypriots and Turkish Cypriots is shown. Armenian Cypriots have lower genetic differentiation with Greek Cypriots and Turkish Cypriots, compared with the Maronite Cypriots who have moderate differentiation (Fig 1).

### Y-haplotypes shared among Cypriots and populations of interest

Using the data from the current study and other publications, the number and percentage of individuals who carry a number of Cypriot haplotypes (Greek Cypriots, Turkish Cypriots, Maronite Cypriots and Armenian Cypriots) were calculated. The percentage of shared haplotypes represent the number of individuals, calculated also as a percentage, who have an exact match with the 17 Y-STR haplotype in each Cypriot group. As mentioned above, Maronite Cypriots share a number of haplotypes between them and Armenian Cypriots (S4 Table). The highest percentage of shared haplotypes is observed between Greek Cypriots and Turkish Cypriots. Maronite Cypriots share haplotypes with Greek Cypriots (~5.4%), Syrians (3.6%), Turkish Cypriots (~1.8%) and Lebanese (~1.8%) (Table 4). The most shared haplotypes between Armenian Cypriots and the eight specified populations are observed with the Armenian population (~20%). A small number of shared haplotypes were also observed between Armenian Cypriots and Turkish Cypriots (~4%) (Table 4).

**Table 4:** Number and percentage of shared haplotypes between & within Cypriots and populations of interest.

Population	Cyprus [Maronite Cypriots]	Cyprus [Armenian Cypriots]	Cyprus [Greek Cypriots]	Cyprus [Turkish Cypriots]
Cyprus [Maronite Cypriots]	1 (26.79%)	0 (0.0%)	3 (0.92%)	7 (1.91%)
Cyprus [Armenian Cypriots]	0 (0.0%)	10 (18.52%)	0 (0.0%)	1 (0.27%)
Cyprus [Greek Cypriots] [15]	3 (5.36%)	0 (0.0%)	59 (18.04%)	40 (10.90%)
Cyprus [Turkish Cypriots] [15]	1 (1.79%)	2 (3.70%)	52 (15.90%)	78 (21.25%)
Armenia [Armenians] [8]	0 (0.0%)	11 (20.37%)	0 (0.0%)	6 (1.63%)
Egypt [Egyptians] [9]	0 (0.0%)	0 (0.0%)	0 (0.0%)	1 (0.27%)
Syria [Syrians] [10]	2 (3.57%)	0 (0.0%)	9 (2.75%)	3 (0.82%)
Lebanon [Lebanese] [12]	1 (1.79%)	0 (0.0%)	8 (2.45%)	24 (6.54%)
Turkey [Turkish] [11]	0 (0.0%)	0 (0.0%)	0 (0.0%)	0 (0.0%)

### Pairwise genetic distance (Rst) between Cypriots and populations of interest

Additional pairwise genetic distances according to the Y-haplogroup paths were calculated using YHRD online tool (S5 Table, S1 Fig). The criterion for the selection of the countries in the calculation was the distribution, the origin and the Y-path of all the assigned haplogroups in the Cypriot population (S6 Table). Each haplogroup originated in specific regions and through various historical events, they expanded to the countries where they are frequent today.

Maronite Cypriots were very close genetically with Ethiopia (Tigray) and Egypt (Assiut), with genetic distances between 0–0.05. Moderate genetic differentiation was observed among Maronite Cypriots and populations from the following countries/regions: Albania, Azerbaijan, Bahrain, Lebanon, Northern Region of Egypt, Iran, Iraq, Israel and regions under the Palestinian Authority, Democratic Republic of Congo, Mediterranean Region of Turkey, Northern Israel, Russia and Yemen (S5 Table).

Armenian Cypriots were shown to have low genetic differentiation with more populations in contrast to the Maronite Cypriots. Armenian Cypriots were demonstrated to be genetically closer to Central Anatolia, Mediterranean and Southeastern Anatolia Turkey, Iran and Iraq with Rst values lower than 0.01. Low genetic differentiation between Armenian Cypriots was observed with populations from countries/regions of: Albania, Azerbaijan, Bahrain, Bulgaria, Democratic Republic of Congo, Italy, Northern Iraq, Northern Israel, Lebanon, Romania and Yemen (S5 Table). Moderate genetic differentiation between Armenian Cypriots was shown with populations from a number of countries/regions such as: Afghanistan, Central African Republic, Bosnia and Herzegovina, Cameroon, China, Croatia, Israel and Palestinian Authority Area, Jordan, Slovenia, Russia, Yemen, Serbia, Libya, Mongolia, Northern Egypt and the region of Turkey facing the Aegean Sea.

**Fig 2:**
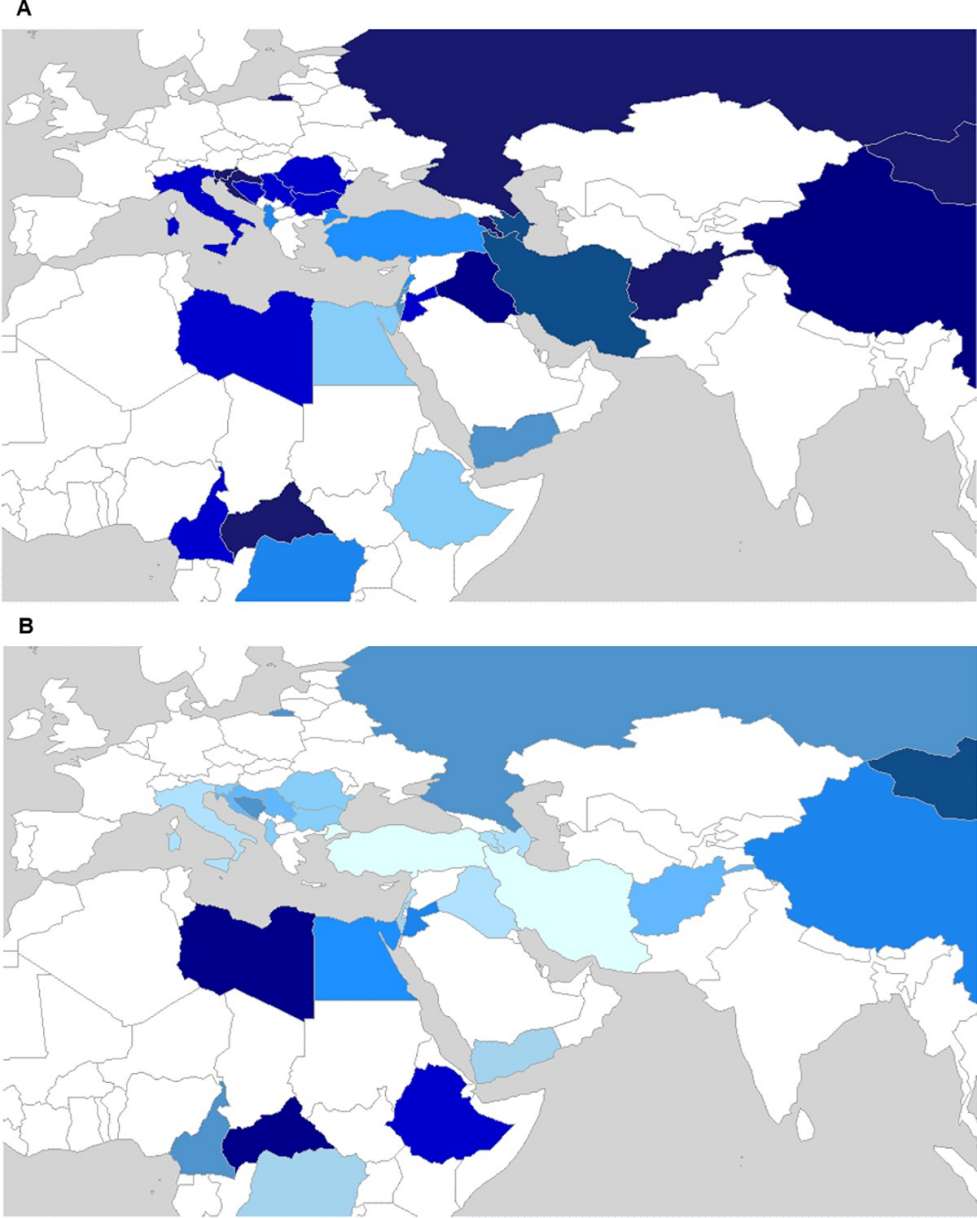
Contour maps based on the additional calculated pairwise genetic distances. (A) Pairwise genetic distances compared with Maronite Cypriots (B) Pairwise genetic distances compared with Armenian Cypriots. The colour scale indicates the range of the Rst values; the lower the colour intensity is, the lower the genetic differentiation between populations. Light blue indicates low genetic differentiation with values in the range 0–0.05 while dark blue indicates great genetic differentiation with values greater than 0.15.

In order to visualize the genetic correlations of Maronite Cypriots and Armenian Cypriots with the aforementioned countries, contour maps were created based on additional calculations, using R programming and R studio (Fig 2).

### Y-haplogroup analysis

Eight major Y-haplogroups: E, G, I, J, R, L, Q and T with their subclades within the major haplogroups were identified. Most of the samples were assigned to their predicted haplogroup, in accordance with the Y-DNA Haplogroup Predictor NEVGEN tool, with the exception of 6 samples out of the 113, which were assigned to a different haplogroup. Predicted and assigned Y-haplogroup for each individual is shown in S2 Table. The percentage error of the predicted Y-haplogroup was 5.31%.

Y-haplogroup frequencies were calculated separately for each population, Maronite Cypriots and Armenian Cypriots. All 113 samples were assigned to major haplogroups and Y-haplogroup subclades. As shown in Table 5, the most frequent Y-haplogroup in Maronite Cypriots was the Y-haplogroup J, subclade J2a-L559 (26.32%). The second most frequent Y-haplogroup was E, subclade E1b1b1-M123 (21.05%) followed by Y-haplogroup J1-M267 with a frequency of 14.04%. Additional Y-haplogroups are found but at lower frequencies. Most of the Armenian Cypriots were assigned to Y-haplogroup R1b-M343/M415 (28.57%). Y-haplogroup J2a-L559 is found also at a high frequency in Armenian Cypriots (25%). A significant number of individuals was assigned to Y-haplogroup G2-P287 (10.71%) (Table 5).

**Table 5:** Number and percentage of Y-haplogroups and subclades in each population group.

Y-haplogroup	Maronite Cypriots	Armenian Cypriots
**E1a-M132**	2 (3.51%)	n/a
**E1b1b-M35**	2 (3.51%)	1 (1.79%)
**E1b1b-M35.1**	1 (1.75%)	n/a
**E1b1b-M78**	1 (1.75%)	n/a
**E1b1b-V22**	2 (3.51%)	n/a
**E1b1b-M123**	12 (21.05%)	1 (1.79%)
**F-M89**	1 (1.75%)	n/a
**G2a1-Z6552**	n/a	2 (3.57%)
**G2a2-M3308**	1 (1.75%)	4 (7.14%)
**I2-L68**	1 (1.75%)	2 (3.57%)
**J-P209**	1 (1.75%)	n/a
**J1-M267**	1 (1.75%)	n/a
**J1a-Z2392**	7 (12.28%)	5 (8.93%)
**J2a-L559**	15 (26.32%)	14 (25%)
**J2b-M12**	1 (1.75%)	2 (3.57%)
**Q2-F1280**	n/a	1 (1.79%)
**R1a-M420**	n/a	2 (3.57%)
**R1b-M343**	n/a	1 (1.79%)
**R1b-L389**	4 (7.02%)	15 (26.79%)
**R2-M479**	n/a	3 (5.36%)
**T-M184**	3 (5.26%)	2 (3.57%)
**T1-PF5641**	2 (3.51%)	1 (1.79%)

Differences were observed when comparing the Y-haplogroup frequencies between Maronite Cypriots and Armenian Cypriots. Y-haplogroup E, subclade E1b1b-M123 was found at a high frequency (21.05%) in Maronite Cypriots, while in Armenian Cypriots it was found at a very low frequency (1.79%). Similarly, in Armenian Cypriots Y-haplogroup R1b-M343/M415 was found at a high frequency (26.79%) whereas in Maronite Cypriots it was observed at a much lower frequency (7.02%). Haplogroups J1a and J2a appeared at similar frequencies in the Maronite and Armenian Cypriots (12.28 vs 8.93%) and (26.32 vs 25%) respectively. In the remaining haplogroups the number of observations were low (0–3%) to allow any accurate deductions on relative frequencies.

**Fig 3:**
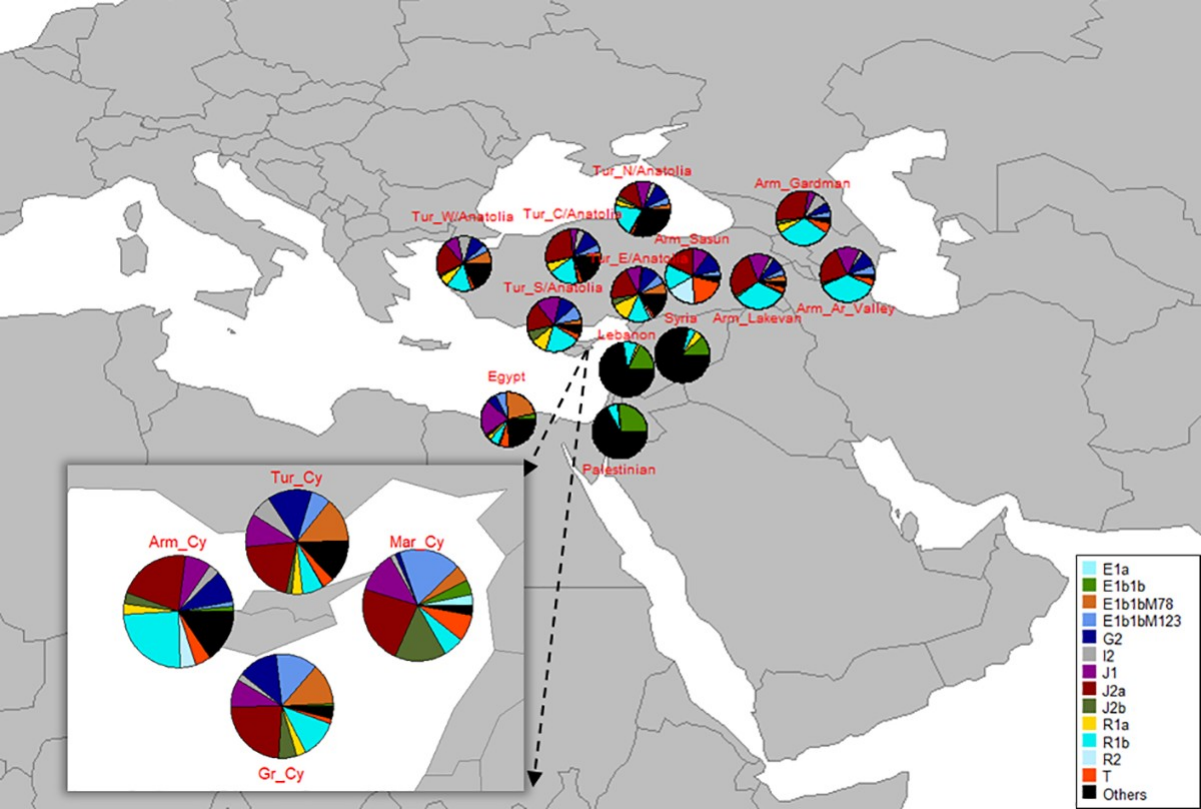
Y-haplogroup frequencies of all Cypriots and all specified populations in the current study. The zoom-in window shows all the Y-haplogroup frequencies among all Cypriots in pie charts. The dark red color, which corresponds to Y-haplogroup J2a, was common in all Cypriots, Armenians and Turks, while it was not observed in Palestinian, Lebanese, Egyptian and Syrian populations. Y-haplogroup R1b was observed in all populations but in different frequencies.

Y-haplogroup major subclade J2a-L559 is the most frequent Y-haplogroup among all Cypriots with frequencies greater than 20%. As shown in Fig 3, J2a-L559 is found at approximately the same frequency in all Cypriots. Y-haplogroup subclade E1b1b-M123 is frequent in both Maronite Cypriots and Greek Cypriots (21.05%, 13.10%). Y-haplogroup R1b-M343/M415 is frequent in both Armenian Cypriots and Greek Cypriots (28.57%, 11.90%), as well as, Y-haplogroup subclade G2 with frequencies 10.71%, 12.50%, 14.20%, in Armenian Cypriots, Greek Cypriots and Turkish Cypriots, respectively (S7 Table). Y-haplogroup subclade E1b1b-M78 is found in Maronite Cypriots, Greek Cypriots and Turkish Cypriots only (5.26%, 12.80%, 13.90%, respectively). Furthermore, J1-M267 is found in all Cypriots but not at a high frequency, with values between the range of 8–14% (S7 Table).

Y-haplogroup frequencies from Armenia, Egypt and Turkey from which Armenian Cypriots originated as well as Lebanon and Syria from which Maronite Cypriots originated, were used for comparative analyses with all Cypriots [8–10, 15]. Y-haplogroup frequencies of all populations can be found in S7 Table. Mappies according to the haplogroup frequency of each population can be seen in Fig 3. The haplogroups considered in the analysis, are those, which are common in all Cypriots. Y-haplogroup J2a-L559 was the most frequent haplogroup in all Cypriots. This major subclade was found at a high frequency in all Armenian populations (Lake Van, Sasun, Ararat Valley, Gardman) and in most Turkish populations (Central, North, South and West Anatolia) [frequencies between 17–30%].

R1b-M343/ M415, which was the most frequent Y-haplogroup subclade in Armenian Cypriots, was found at a high frequency in all Armenian and Turkish populations (Fig 3). Thus, the genetic correlation based on the pairwise genetic distances was also reflected in the Y-haplogroup frequencies. Y-haplogroup subclade E1b1b-M35 was one of the most frequent haplogroups among Maronite Cypriots and was found at a high frequency in Lebanese and Palestinian populations with frequencies greater than 15%. Subclade E1b1b-M78 was found at a high frequency in Egyptians, which was observed also in Maronite Cypriots.

### MJN haplogroups

**Fig 4:**
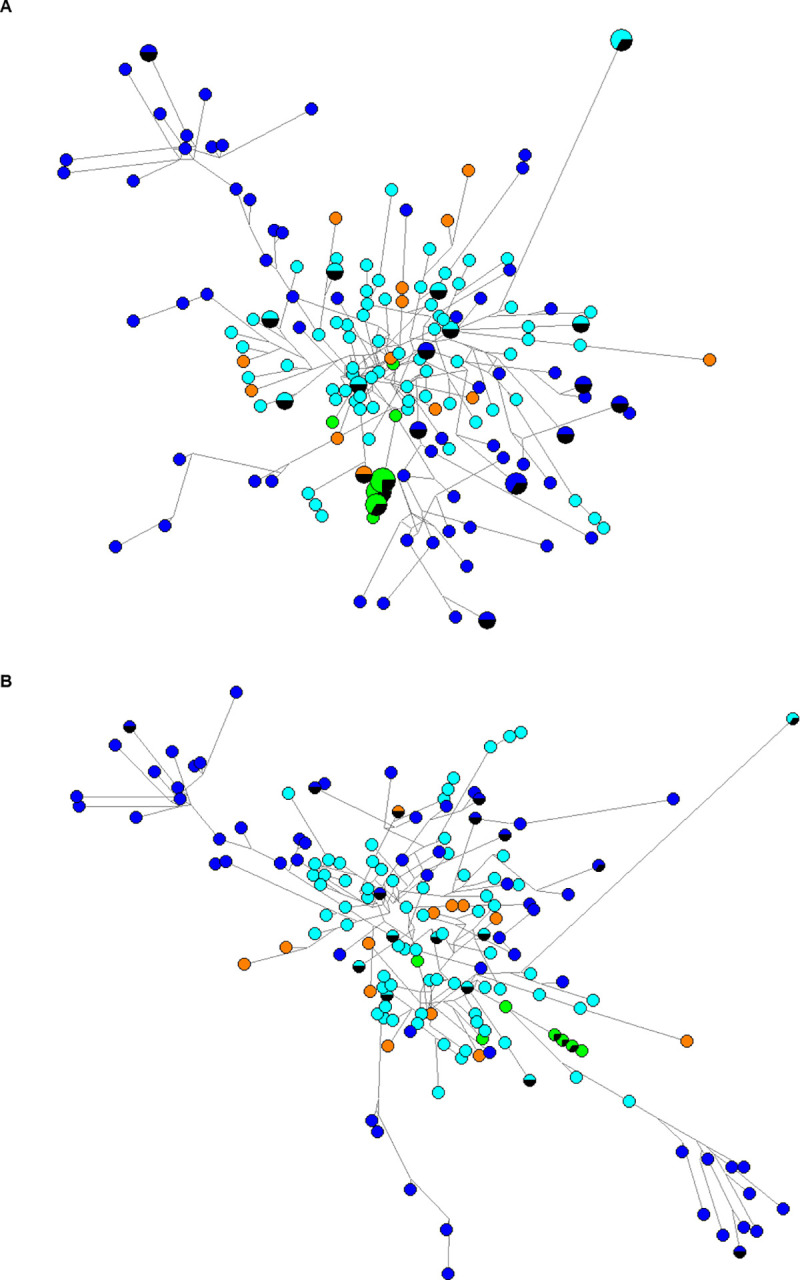
Median joining networks of all Cypriot haplotypes, which were assigned to Y-haplogroup J2a. (A) MJN among all Cypriot 17 Y-STR haplotypes assigned to Y haplogroup J2a. (B) MJN among all Cypriot haplotypes after the Star contraction option. MJN after the star contraction option is more simplified and expansion events are easy to be analyzed. The edges (lines) represent the mutation distance between individuals and the nodes (circles) represent the frequency of each haplotype. Two constructed networks are shown, where the dark blue indicates Turkish Cypriots, light blue Greek Cypriots, green Maronite Cypriots and orange Armenian Cypriots haplotypes.

MJN analysis was performed on the Greek Cypriot, Turkish Cypriot, Maronite Cypriot and Armenian Cypriot 17 Y-STR data which were previously assigned to the Y-haplogroup J2a-L559 (Fig 4). It is known that haplotypes which belong to the same haplogroup share a common ancestor, therefore, all haplotypes which were assigned to the only common haplogroup among all Cypriots, J2a-L559, were used in the MJN analysis for the age calculation of their common ancestor and subsequently, to estimate the expansion time of the haplogroup in the Cypriot population. The first MJN (Fig 4A) shows the correlation of all 17 Y-STR haplotypes which were assigned to J2a-L559. This network shows that all Cypriots in this Y haplogroup are very close to each other, since there are not many mutational steps between them. It is also observed, that a number of haplotypes are shared between Cypriots and this is explained by the node size. Node size is proportional to the number of shared haplotypes. The smallest nodes represent only one haplotype and the larger nodes represent more than one haplotype and thus, shared haplotypes.

MJNs based on Y-haplogroups, were constructed before (Fig 4A) and after the application of the star contraction option (Fig 4B), to see if the option created a star cluster between a haplogroup. The network in Fig 4B, shows the network after the star contraction option. The clusters characterize different historical events, which occurred in the population and can be estimated chronologically using the time estimation option of the Network 4.6.1.6, Fluxus Engineering [23, 24]. Two historic demographic expansion events were created among the haplotypes. The first expansion event includes Greek Cypriot, Turkish Cypriot and Armenian Cypriot haplotypes while the second expansion event includes at least one haplotype from each of the four Cypriot groups (Fig 4B).

**Fig 5:**
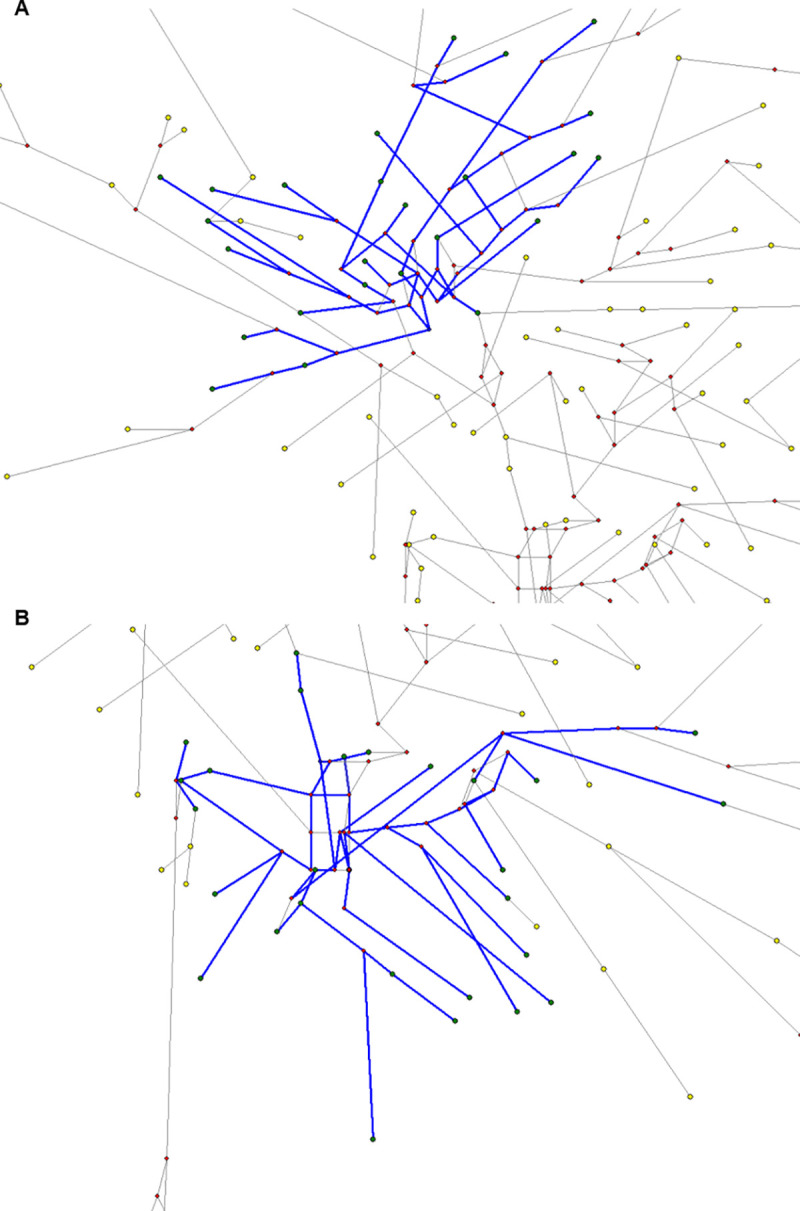
Estimation of the time of expansion events between all Cypriots. (A) First expansion event among Armenian Cypriots, Greek Cypriots and Turkish Cypriots, (B) Second expansion event between all Cypriots. Yellow circles represent the Y-haplotypes, the red circles represent the median vectors and the blue lines are the Y-haplotypes, which consist the expansion events.

Y haplogroup J2a was introduced to Cyprus during the Late Bronze Age and Early Iron Age, approximately around 1050 BC. Both first and second expansion events between Armenian Cypriots, Maronite Cypriots, Greek Cypriots and Turkish Cypriots were estimated to have occurred 3066 years ago +/- 407 SD or 3066 +/- 476 SD (Fig 5A).

An alternative method was used to estimate the time of expansion. From both MJNs and expansion events, the expansion time was estimated 3136 years ago. Confidence intervals were calculated for both expansion events to be, 3136 +/- 1196 SD and 3136 +/- 934 SD, respectively. The above data from the two methods used for the expansion time calculation, support the statements based on Voskarides et al. (2016) and Heraclides et al. (2017) namely, that the Y haplogroup J2a was spread during the Late Bronze Age [15, 27].

## Discussion

### Shared paternal ancestry among Greek Cypriots, Turkish Cypriots, Armenian Cypriots and Maronite Cypriots

Y-STR haplotypes from Armenian Cypriots, Greek Cypriots, Maronite Cypriots and Turkish Cypriots were compared to find the genetic differentiation or any similarities between them. The common paternal ancestry between Greek Cypriots and Turkish Cypriots has been reported in the study by Heraclides et al. (2017) [15]. The present study is an extension of this previous work, to investigate further the ancestry of the Cypriot population. Results based on the Y-chromosome haplotypes showed that the two main population groups of the current study, that is, Maronite Cypriots and Armenian Cypriots, do not share a large number of Y-STR haplotypes between them, and thus, show a moderate (0.05–0.15) genetic differentiation between them. Armenian Cypriots are genetically closer to Greek Cypriots and Turkish Cypriots compared to the Maronite Cypriots who have a moderate genetic differentiation with Greek Cypriots and Turkish Cypriots.

Based on the inter-population analysis, Maronite Cypriots are genetically closer to the Lebanese population; this was an expected finding since in the history of Cyprus, it is documented that during the different waves of Maronites coming to Cyprus, most of them were from Lebanon. Moderate genetic differentiation is shown between Maronites and Egyptians, Israelis and Palestinians. Another reason could be that samples from different regions of Syria were used. No significant correlation was noted between Maronite Cypriots and Armenian and Turkish populations since the Rst values were greater than 0.15. Armenian Cypriots show very low genetic differentiation between Turks and Syrians. The genetic correlation between Armenian Cypriots and Turks is explained by the origin of many Armenian Cypriot samples in this study, which were from different regions of present-day Turkey (Adana, Istanbul, Silifke and Cilicia). Additionally, Armenian Cypriots are genetically close with Lebanese and Israeli populations with Rst values lower than 0.05. The correlations between Turks, Lebanese and Israelis were expected because a number of Armenians came to Cyprus from these three countries. There is no noted history with regards to the relationship of Armenian Cypriots and Syrians.

The most frequent haplogroup in Maronite Cypriots was Y-haplogroup J in major subclade J2a-L559 (23.80%). This subclade was common also among all the other Cypriots (Armenian Cypriots, Greek Cypriots and Turkish Cypriots) and was observed at high frequencies (20–27%). J2a-L559 was found with a high frequency in all Armenian and Turkish populations, except in those populations of North Anatolia in Turkey. In Lebanon, Syria and Palestine the subclade was absent, but in Egypt it was found at a very low frequency (~2%).

The subclade E1b1b-M123 was found in Maronite Cypriots at a high frequency (21.05%) (Table 5). The Y haplogroup E was found at a high frequency in Lebanon, Syria, Egypt and Palestine (Fig 3). In Anatolian and Caucasian Armenias it was present at a very low frequency (~5%). Considering the fact that Maronite Cypriots originated from Lebanon and Syria it was expected that haplogroup E would be frequent in those two countries. Regarding haplogroup R and subclade R1b-M343/M415, it is frequently observed in Lake Van, Ararat Valley and Gardman (~30%), Sasun (15%) and in all regions of Turkey (~15–20%). This subclade appears also in Egypt, Lebanon, Syria and Palestine, but at a lower frequency (3–8%) than in the other populations [9, 10]. Armenian Cypriots originated mainly from Armenia and present-day Turkey, where in these regions haplogroup subclade R1b-M343/M415 appears at a high frequency.

Previous studies [8, 36] on the Y-chromosome haplotypes and haplogroups regarding Armenian populations, did not focus on the Armenian Cypriot population. The most prevalent haplogroup for the Armenian populations was R in the lineage R1b1b1 (R-L23) and haplogroup J in the lineage J2a (J-M410, J-M172) [8]. Comparing the study by Herrera et al. (2012) [8], with the results of the haplogroup analysis of the Armenian Cypriots of the current study, an agreement is observed regarding the prominent haplogroups. Armenian populations have a high frequency of haplogroup subclade R1b1a2a (R-L23), whereas haplogroups E, G and J were found at lower frequencies (5%). Hovhannisyan et al. (2014), examined six Armenian populations and haplogroup subclade R1b1a2 was the most prevalent among all [36].

Y haplogroup J1-M267 was found in all Cypriots with frequencies between the range 9–15%. Previous studies [37–39] found a correlation between allelic variants at the DYS458 locus and the Y haplogroup J1-M267. This is confirmed also seen in the current study since both Maronite Cypriots and Armenian Cypriots who were assigned to J1-M267, do have the DYS458.2 allelic variant. Furthermore, in 17 Y-STR haplotypes from Greek Cypriots and Turkish Cypriots, all Greek Cypriots and 37 out of 39 Turkish Cypriots have the DYS458.2 variant and were assigned to the specific subclade.

### Genetic distances between populations

Comparing the Y-STR haplotypes with the data of the countries: Armenia, Egypt, Israel, Lebanon, Syria, Turkey and the calculated Rst, it was observed that Maronite Cypriots showed low genetic differentiation with a value between 0–0.05 with Lebanese and moderate differentiation with Egypt, Syria, Israel and Israel and Palestinian Authority Area populations. The genetic distances between Maronite Cypriots and countries of the Levant showed a low genetic differentiation between Maronites and Egyptians. Populations of Iraq, Israel and Palestinian Authorities and Turkey belong to the Levant and have a moderate genetic differentiation with Maronite Cypriots. Furthermore, subclade E1b1b-M35 was distributed mainly in Ethiopia and was confirmed with the genetic distance calculated for Maronite Cypriots (~0.05).

Caucasian haplogroup J was also frequent in Armenian Cypriots and more specifically, subclade J2a-L559. The lowest genetic differentiation between Armenian Cypriots was observed with Turkey (Mediterranean and South-eastern regions), Iran and Iraq, with values ~0.01. Furthermore, Armenian Cypriots are also genetically closer with populations from Armenia, Azerbaijan, Bahrain, Beirut, Bulgaria, Croatia, Democratic Republic of Congo, Iraq, Iran, Israel, Romania, Yemen and Italy. There is also genetic correlation among Armenians and populations from Afghanistan, Central African Republic, Bosnia and Herzegovina, Cameroon, China, Croatia, Israeli and Palestinian Authority, Jordan, Slovenia, Russia, Yemen, Serbia, Libya, Mongolia, Northern Egypt and the Aegean Region of Turkey. Some genetic correlations are explained by the origin of J2a as well as subclade G2 that originated from Caucasus. However, haplogroup G was expanded to European populations such as Croatia, Romania, Hungary and Serbia [40]. This explains the genetic relation among Armenian Cypriots with those countries. One unexpected correlation, according to the Rst values, was between Armenian Cypriots and Syrians, since there is no information about their migration from Syria to Cyprus. This correlation may be explained by the distribution of haplogroup J during the Iron Age, to Anatolia and Eastern Mediterranean which includes Turkey, Lebanon, Syria, Egypt and other countries [41].

The MJN analysis revealed two different expansion events among Cypriots. The first event consisted of Armenian Cypriots, Greek Cypriots and Turkish Cypriots and the second event consisted of all four Cypriot groups (Armenian Cypriots, Greek Cypriots, Maronite Cypriots and Turkish Cypriots). According to previous studies, J2a-L559 was spread from the Caucasus to Anatolia, Greece and then to Cyprus, during the Late Bronze Age [27, 42–44]. The expansion time calculations showed that the two events for all the Cypriots pooled together, occurred 3066 or 3136 years ago, depending on the method used. This result was expected, since all Y-STR haplotypes that were used for the expansion time calculation have been assigned to the same Y-haplogroup J2a. This time-frame coincides in the Late Bronze Age and thus, confirms the previous observations for the Y-haplogroup subclade J2a-L559 [27].

## Conclusion

The sample size of Maronite Cypriots and Armenian Cypriots in this study was small (57 Maronite Cypriots and 56 Armenian Cypriots). This is a limitation of this study, but it should be noted that these samples, assuming that half of these populations are males, are estimated to constitute 1.63% and 3.73% of the Maronite Cypriot and Armenian Cypriot male population, respectively. More importantly, despite the small sample size of the participants, the Y haplogroup frequencies observed in this study are consistent with previous studies on non-Cypriot Maronites and Armenians [6–8, 45]. Lastly, the estimated time-lines agree with the history of the settlement of Maronite Cypriots and Armenian Cypriots in Cyprus. In view of all the above, the Armenian Cypriot Y haplogroups presented in this study, for the first time, most likely reflect correctly the distribution of the major Y haplogroups in this group.

The only common haplogroup identified between all Cypriots in this study, is haplogroup J2a-L559. The subclade is characterized as mainly Caucasian [46] although, it was expanded to many regions during the ancient years. During the Paleolithic period between 22000–15000 years ago, it was found in the Middle East and was expanded to Europe. In the Mesolithic period it was found in Caucasian and the Caspian Region, where it was distributed to Mesopotamia and the Levant, between 2500–1050 BC [46]. In Anatolia and the Eastern Mediterranean, it was expanded by the end of the Bronze Age and by the early Iron Age. In this study, two population expansion events of haplogroup J2a were calculated and occurred 3066 and 3136 years ago. These results confirmed the findings from other studies on the Cypriot population with respect to the expansion time of haplogroup J2a (J-M12) [15, 27], estimated in the Late Bronze Age and Early Iron Age (1045 BC or 1115 BC, depending on the method used).

## Supporting information

S1 Fig Y path of all Y haplogroups which are found in Maronite and Armenian Cypriots. (TIF)

S1 Table Y SNP table and sequencing primers used for Y SNP genotyping. (XLSX)

S2 Table Y STR haplotypes of Maronite and Armenian Cypriots. For each haplotype, predicted and assigned haplogroups are shown and the methodology used for the Y-SNP determination.
(XLSX)

S3 Table Y-STR analysis results in the Maronite Cypriots and Armenian Cypriots. (XLSX)

S4 Table Shared haplotypes among all Cypriots and populations of interest. (XLSX)

S5 Table Additional pairwise genetic distances between Maronite Cypriots and Armenian Cypriots and other countries based on the Y-haplogroup paths. (XLSX)

S6 Table References of YHRD data used for the AMOVA analysis. (XLSX)

S7 Table Y haplogroup frequencies of all Cypriots and specified populations. (XLSX)
