# Correction: Comparative Y-chromosome analysis among Cypriots in the context of historical events and migrations

**DOI:** 10.1371/journal.pone.0267875

**Published:** 2022-04-26

**Authors:** Irene Moutsouri, Anna Keravnou, Panayiotis Manoli, Stefania Bertoncini, Kyriaki Michailidou, Vasilis Christofi, Stavroulla Xenophontos, Marios A. Cariolou, Evy Bashiardes

This article [1] includes a reference error: the wrong article was listed for reference 16. The statements citing reference 16 should have cited the following:

Gurkan C, Sevay H, Demirdov DK, Hossoz S, Ceker D, Terali K, Erol AS. Turkish Cypriot paternal lineages bear an autochthonous character and closest resemblance to those from neighboring Near Eastern populations. *Ann Hum Biol*. 2017; 44(2):164–174. (https://doi.org/10.1080/03014460.2016.1207805) PMID: 27356680

In addition, the article does not include accurate terminology when describing the Syriac samples obtained from refugee camps in Erbil, Iraq. These samples were described in [1] as Syrian, Syria [Syrians] or Syria [Syriacs], whereas, article [2] indicates that these were Syriac samples “mostly collected at various refugee camps in Erbil”. Where samples from the Erbil camps are described in [1], “Syrian”, “Syria [Syrians]” or “Syria [Syriacs]” should be replaced by “Northern Iraq [Syriacs]”.

This pertains to the following in-text citations, summarized below in Table 1:

**Table 1:** In-text corrections.

Article Section	Subheading, Paragraph, sentence	Text in [1]	Replacement Text
Materials and Methods	Haplotype (Y-STR) based analysis, 3, 2	Syrians	Northern Iraq [Syriacs]
Results	Inter-population analysis, 1, 2	Syria	
	Inter-population analysis, 1, 5	Syrians	
	Inter-population analysis, 2, 2	Syrians	
	Y-haplotypes shared among Cypriots and populations of interest, 1, 5	Syrians	
Discussion	Shared paternal ancestry among Greek Cypriots, Turkish Cypriots, Armenian Cypriots and Maronite Cypriots, 2, 9	Syrians	
	Genetic distances between populations, 1, 1	Syria	
	Genetic distances between populations, 2, 6	Syrians	
	Genetic distances between populations, 2, 6	Syria	
	Genetic distances between populations, 2, 7	Syria	
Figures	Figure 1	Syria [Syriacs]	
Tables	Table 3, column 1, row 8 Table 3, column 1, row 22 Table 3, column 3, row 2 Table 3, column 1, row 14	Syria [Syriacs]	
	Table 4, column 1, row 8	Syria [Syrians]	
Supporting Information	S4 Table	Syria [Syriacs]	

Results derived from analyses of the Erbil camp samples cannot be generalized as representing findings about the Syrian population.

The authors apologize to the authors of [2], the Editors and readers of *PLOS ONE* for these errors in the published article [1].
