# Supplementary figures and images for: Comparative Y-chromosome analysis among Cypriots in the context of historical events and migrations
Source: PLoS One. 2021 Aug 23;16(8):e0255140. doi: 10.1371/journal.pone.0255140 (PMC8382168; doi:10.1371/journal.pone.0255140)

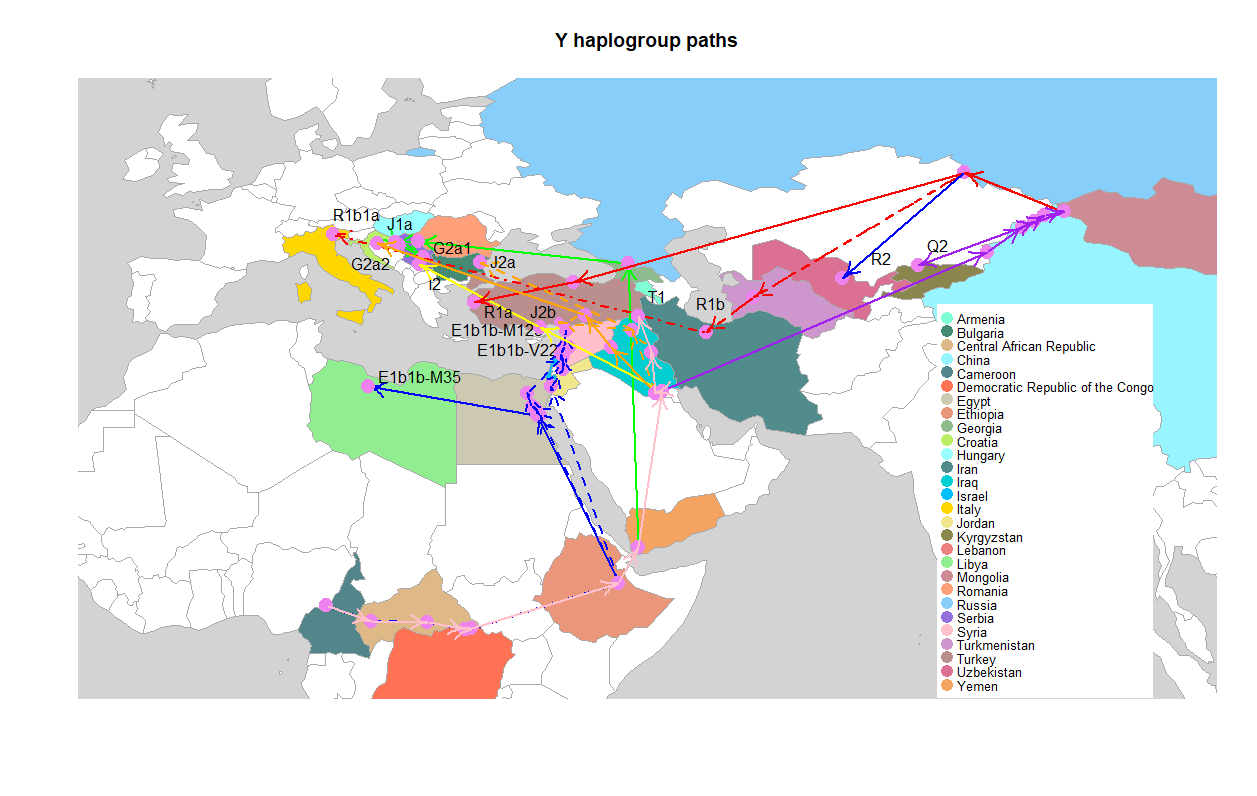

Supplement: S1 Fig — (TIF) [file pone.0255140.s001.tif]
